# VII Brazilian Consensus guidelines for the detection and interpretation of anti-cell autoantibodies on HEp-2 cells

**DOI:** 10.1186/s42358-025-00493-0

**Published:** 2025-11-25

**Authors:** Wilson de Melo Cruvinel, Paulo Luiz Carvalho Francescantônio, Wilton Ferreira Silva Santos, Fabiano de Almeida Brito, Sandra Gofinet Pasoto, Andressa Mathias, Carlos David Araújo Bichara, Diana Landoni, Trischna Martins Berger, Eliane Aparecida Rosseto Welter, Cristóvão Luis Pitangueira Mangueira, Eloisa Bonfá, Henrique Ataíde Mariz, Jozelia Rego, Lisiane Maria Enriconi dos Anjos, Valeria Valim, Antônio Carlos Ximenes, Luis Eduardo Coelho Andrade

**Affiliations:** 1 Pontifícia Universidade Católica de Goiás - School of Medical and Life Sciences, Avenida Universitária 1.440, Setor Universitário, Goiânia, GO, CEP: 74605-010 Brazil; 2 Instituto de Pós-Graduação e Graduação (IPOG), Goiânia, Brazil; 3 Escola Superior de Ciências da Saúde do Distrito Federal (ESCS), Laboratório Sabin, Brasília, Brazil; 4 Universidade Federal de Minas Gerais, Department of Clinical Pathology, School of Medicine, Belo Horizonte, Brazil; 5 Hermes Pardini Group, Vespasiano, Brazil; 6 Reumatologia, Hospital das Clinicas HCFMUSP, Faculdade de Medicina, Universidade de São Paulo, São Paulo, Brazil; 7 Fleury Medicine and Health Laboratory, Immunology Division, São Paulo, Brazil; 8 Centro Universitário Fibra, Amaral Costa Medicina Diagnóstica, Belém, Brazil; 9 Rheumatology Division, Department of Medicine, Federal University of São Paulo, São Paulo, Brazil; 10 Departamento de Patologia Clínica e Anatomia Patológica, Hospital Israelita Albert Einstein, São Paulo, Brazil; 11 Faculdade Israelita de Ciências da Saúde, Sociedade Beneficente Israelita Brasileira Hospital Albert Einstein, São Paulo, Brazil; 12 Rheumatology Department, Federal University of Pernambuco, Recife, Brazil; 13 Faculdade de Medicina, Universidade Federal de Goiás, Goiânia, Brazil; 14 Universidade do Sul de Santa Catarina, Tubarão, Brazil; 15 Universidade do Vale do Itajaí, Itajaí, Brazil; 16 Universidade Federal do Espírito Santo, Hospital Universitário Cassiano Antônio Moraes (HUCAM), Vitória, Brazil; 17 Hospital Geral de Goiânia Alberto Rassi (HGG), Goiânia, Brazil

## Abstract

**Background:**

The VII Brazilian Consensus on Autoantibodies against HEp-2 Cells (BCA/HEp-2), dedicated to the identification of autoantibodies targeting cellular constituents in HEp-2 cells, was held on October 5–6, 2023, in Goiânia (GO, Brazil), as part of the 40th Brazilian Congress of Rheumatology.

**Main body:**

Nineteen researchers and experts from universities and clinical laboratories participated in this meeting, which resulted in important advancements. Among the key updates, two new nuclear patterns were incorporated into the Brazilian classification tree: the nuclear fine speckled with mitotic plate (AC-30) and the myriad discrete speckled pattern strongly associated with antibodies to SS-A/Ro60, previously categorized as AC-4a and now recognized as AC-31. Additionally, the mitotic pattern AC-28 (mitotic chromosomal), characterized by punctate staining of chromosomes in pro- and metaphase stages without interphase cell staining, was included. Adjustments to the classification tree were made to redistribute patterns based on morphological characteristics, harmonize composite pattern designations with ICAP nomenclature, and incorporate the AC-XX group into the classification structure. Guidance was also provided to laboratory analysts on the limitations of identifying patterns with weak reactivity, recommending that reports in such cases be issued according to the appropriate level of analytical competency.

**Conclusion:**

The recommendations aimed to establish clear guidelines for laboratories and clinicians, building upon those from the V and VI Consensuses while further aligning with ICAP recommendations. This consensus marks a significant step forward in adapting ICAP guidelines to the Brazilian context, refining the classification tree and addressing practical challenges faced by the scientific and clinical community.

**Supplementary Information:**

The online version contains supplementary material available at10.1186/s42358-025-00493-0.

## Introduction

The classification and diagnosis of systemic autoimmune diseases are generally based on criteria that include clinical, laboratory, imaging, and histopathological findings, with autoantibodies serving as key markers that play a central role in several of these criteria [1–4]. The indirect immunofluorescence assay on HEp-2 cells (HEp-2 IFA), historically referred to as the antinuclear antibody (ANA) test, enables the detection of a broad spectrum of autoantibodies and is highly relevant in the investigation of certain systemic autoimmune diseases [5]. However, since its initial implementation, several issues have been reported regarding the lack of harmonization in both technical procedures and nomenclature, along with considerable concern about the reproducibility of results across different laboratories [6, 7].

Initiatives to standardize ANA assay on HEp-2 cells in Brazil began in 2000 in the city of Goiânia [8, 9]. The main focus was the development of a classification algorithm for HEp-2 IFA patterns, as well as the establishment of technical guidelines, criteria for test interpretation and reporting, and general recommendations for the test procedure [8, 9]. Over the past two decades, several other topics have been gradually addressed, with emphasis on the refinement of pattern classification, the investigation of their clinical relevance, and the improvement of quality control measures in the assay [9–11].

In 2015, the first ICAP (International Consensus on ANA Patterns) workshop was held in São Paulo during the 12th International Workshop on Autoantibodies and Autoimmunity. As a result, 28 HEp-2 IFA patterns were defined, and each pattern was assigned an alphanumeric code (AC, for anti-cell) [7]. To date, 32 patterns have been recognized [12]. ICAP provided globally applicable guidelines, which were subsequently adapted and incorporated into the recommendations of the Brazilian Consensus on ANA (BCA), enabling a more rational use and progressive enhancement of the HEp-2 IFA test [13, 14]. For example, the fifth edition of the BCA, which incorporated ICAP advancements, also reinforced previous recommendations regarding quality control, emphasizing the need for quality assessment practices to ensure high standards of accuracy and efficiency [13]. This was further expanded in the sixth edition of the BCA/HEp-2, which focused on the harmonization process with ICAP and addressed several key topics related to pattern classification with clinical implications for test interpretation. These included the discussion of complex patterns, the classification of the pattern suggestive of anti-SS-A/Ro60 autoantibodies, and the recommendation for mandatory reporting of nuclear envelope as a separate cell domain. The latter intended to guarantee that analysts would not miss the observation of the nuclear envelope [14].

Since its inception in 2000, the BCA, in close alignment with the ICAP, has had a measurable impact on laboratory performance in recognizing HEp-2 IFA patterns. In a recent nationwide evaluation involving 64 laboratories, accuracy for negative samples (AC-0) reached 95.2%, and most competent-level patterns, such as AC-3 (94.1%) and AC-4/5/31 (94.6%), were also correctly classified at high rates, underscoring the effectiveness of these guidelines in standardizing practice [15]. Moreover, recognition of expert-level patterns averaged 69.7%, demonstrating tangible advances in harmonization. Nonetheless, significant challenges remain, particularly in the correct identification of nuclear patterns with positive metaphase plates (e.g., AC-1, AC-2, AC-29), as well as certain cytoplasmic and mitotic patterns, where accuracy ranged from 20% to 62%. These findings highlight that, while BCA recommendations have demonstrably improved test performance across Brazilian laboratories, ongoing education and reinforcement are still required to overcome persisting difficulties in more demanding pattern categories [15].

The 7th edition of the BCA/HEp-2 incorporated the new patterns recently included by ICAP [12], namely the nuclear fine speckled with mitotic plate (AC-30) and the nuclear myriad discrete speckled (AC-31). It also included the mitotic chromosomal pattern (AC-28), and the unclassified patterns (AC-XX). In addition, it introduced updates to the classification tree at the competent levels and provided guidance for laboratories on the classification of patterns in serum samples with low reactivity. Finally, the seventh BCA/HEp-2 also included an update to the classification tree at the expert level. These updates are highly relevant for clinicians who interpret the test and support the alignment of Brazilian clinical laboratories with the most recent international recommendations.

## Methods

On October 5–6, 2023, during the 40th Brazilian Congress of Rheumatology held in Goiânia (GO, Brazil), nineteen experts in the HEp-2 IFA test from university centers and clinical laboratories across different regions of the country participated in a workshop dedicated to discussing and approving the new BCA/HEp-2 recommendations. The group addressed key challenges through literature reviews, data presentations, and in-depth discussions, culminating in the approval of all agenda items either by consensus or open voting, if necessary. The discussion topics were preselected based on comments and suggestions previously submitted to the BCA/HEp-2 team.

The session began with presentations by Trischna Martins Berger, who shared preliminary data from the ICAP-endorsed HEp-2 CIC project (Clinical and Immunological Characterization of HEp-2 IFA Patterns); Diana Landoni, who presented the results on an ongoning study on the consistency of associations between patterns and specific autoantibodies; Luis Andrade, who addressed the limits in applying consensus recommendations for pattern definition, particularly in low-reactivity sera; Wilson Cruvinel, who presented a proposal on the inclusion of the AC-28 pattern in the Brazilian Consensus classification tree; Paulo Francescantonio, who explored the challenges in characterizing nuclear patterns in the presence of nuclear envelope reactivity; Lisiane Vieira, who presented results from an ongoing study on the Non-DFS (FS/non-DFS) nuclear speckled pattern with stained metaphase plate as a variant pattern with distinct immunological and clinical associations; and again, Wilson Cruvinel, who presented a proposal on the incorporation of the AC-30, AC-31, and AC-XX patterns into the Brazilian Consensus classification tree.

These presentations provided the foundation for the subsequent discussions, which were coordinated by Paulo Francescantonio de Carvalho and Wilson de Melo Cruvinel. Prior to the meeting, all CBA members had reviewed relevant published literature related to the agenda items, although no formal systematic review process was conducted. After thorough debate, a consensus was reached on each topic with no need for open voting. The final recommendations included [1] the inclusion of new patterns recognized by the ICAP into the CBA classification tree [2], the novel reformulated CBA tree accommodating the new patterns in harmonization with the latest ICAP classification tree, and [3] guidance for laboratories on the classification of patterns in low-reactivity serum samples.

### Updates and recommendations

## Inclusion of the nuclear fine speckled with mitotic plate pattern (AC-30)

At the seventh ICAP meeting, the nuclear fine speckled pattern with mitotic plate (NFS with plate) was included in the classification tree, receiving the AC-30 code [12]. The Brazilian Consensus has likewise incorporated this new pattern into its classification tree. It is a distinct pattern that represents a variation of the dense fine-speckled nuclear pattern (AC-2). The AC-30 pattern is characterized by a fine speckled nuclear fluorescence that stains the interphase nuclei and the chromosome metaphase plate. The distribution of speckles is uniform, and the nuclear border is regular [16]. The traditional and well-known AC-2 pattern is characterized by speckles that vary in size, brightness, and density. This heterogeneity includes fine, dim speckles interspersed with coarser and brighter ones, as well as regions with sparse distribution of speckles intermingled with more densely packed areas. As a result of this variability, the nuclear periphery displays an irregular and discontinuous rim. The positive chromosome metaphase plate appears brighter than the interphase nucleus while retaining the same morphological characteristics [17], an aspect not observed in AC-30 [12].

**Fig. 1:**
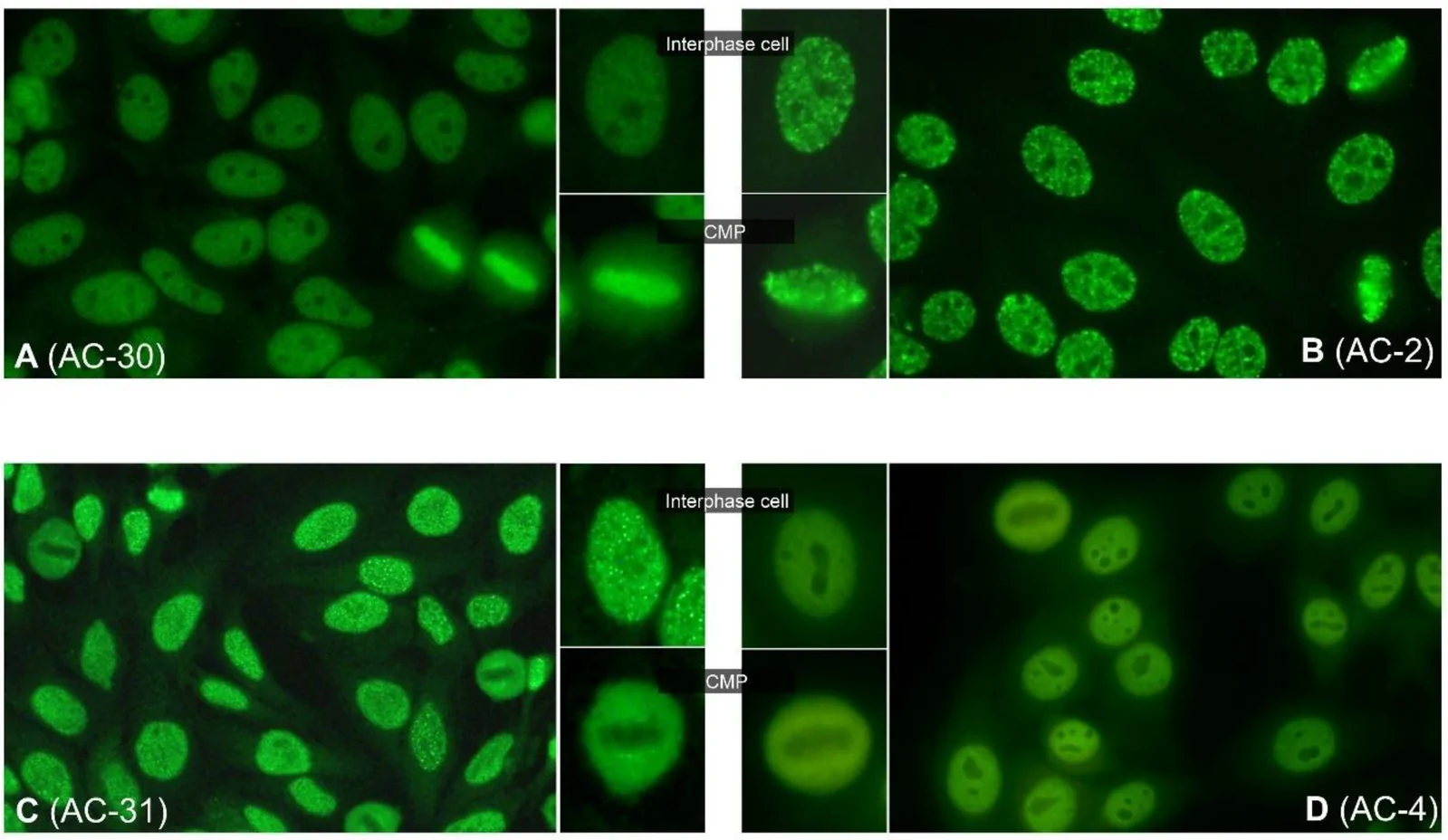
Morphological details of the nuclear patterns. (**A**). Nuclear fine speckled with mitotic plate (AC-30). (**B**). Nuclear dense fine speckled (AC-2). (**C**). Nuclear myriad discrete speckled (AC-31). (**D**). Nuclear fine speckled (AC-4). For each image, an enlarged view of both the interphase cell and the chromosomal metaphase plate (CMP) is displayed at the center. Image credits from the International Consensus on ANA Patterns (ICAP): AC-30 (Werner Klotz & Manfred Herold), AC-2 (John William & Edward Chan), AC-31 (Luis Eduardo Coelho Andrade), AC-4 (Wilson de Melo Cruvinel). (Microscope magnification: 400×)

There is a strong association of the AC-2 pattern with anti-DFS70 antibodies [18–21]. The genuine AC-2 pattern is readily observed when anti-DFS70 is the only antinuclear antibody in the sample, as the presence of additional antinuclear antibodies may obscure the AC-2 characteristics [21]. The monospecific anti-DFS70 antibody, reflected by the AC-2 pattern, is characteristically not associated with systemic autoimmune diseases such as systemic lupus erythematosus [12]. The importance of characterizing the AC-30 pattern lies in its differentiation from the AC-2 pattern, as AC-30 is not associated with monospecific anti-DFS70 antibodies. Moreover, nearly one quarter of sera presenting the AC-30 pattern recognize one or more clinically relevant antinuclear antibodies, including those against dsDNA, nucleosome, Sm, U1-RNP, Scl-70, SS-A/Ro, or SS-B/La [12]. Figure 1A shows the morphological characteristics of the AC-30 pattern. For comparison and to facilitate differentiation from AC-2, an image of the AC-2 pattern (nuclear dense fine speckled) is presented in Fig. 1B.

**Fig. 2:**
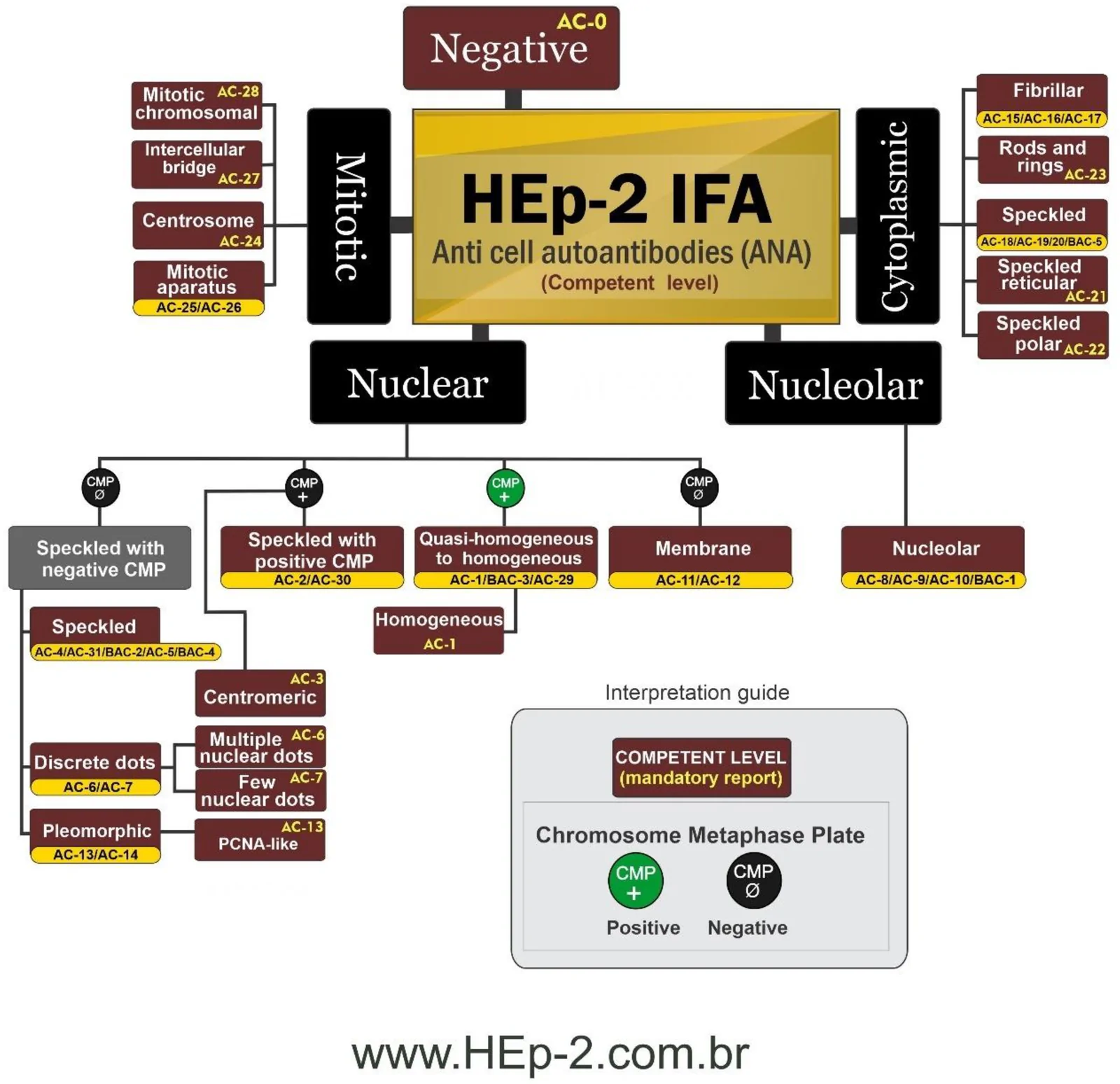
BCA Classification tree with emphasis to the competent-level patterns. AC - Anti-cell antibody patterns classified according to the International Consensus on ANA Patterns (ICAP) [7]. BAC - Brazilian anti-cell antibody patterns referring to transient code used for patterns not yet categorized by ICAP [13]

**Fig. 3:**
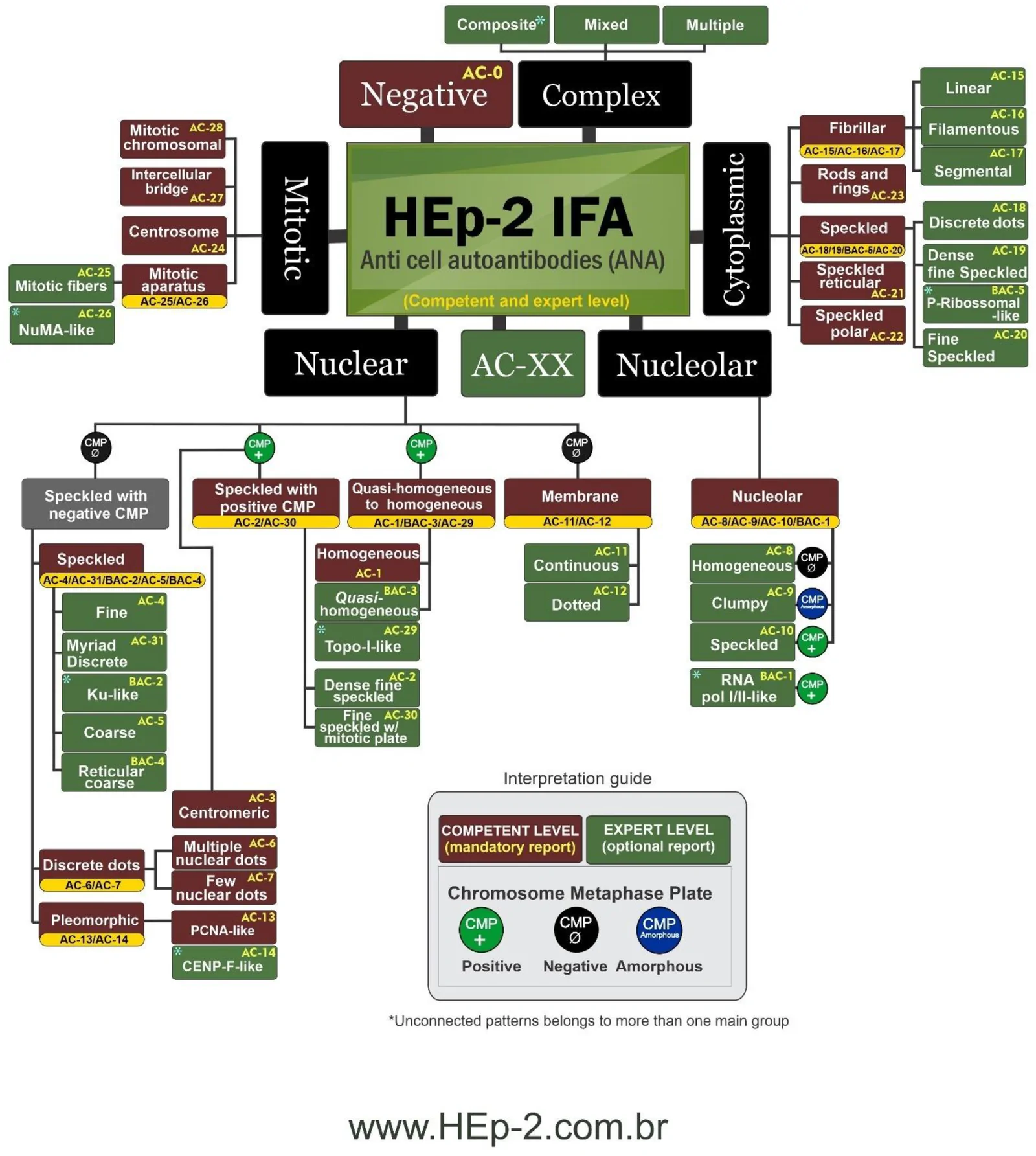
BCA classification tree highlighting the expert-level patterns (optional report). Each expert-level pattern is nested within a corresponding competent-level pattern, allowing the analyst to choose between the expert- and competent-level designations based on the characteristics observed in each sample image. An asterisk indicates that the pattern belongs to the composite group and is therefore not linked to a single main group (AC-14, AC-26, AC-29, BAC-1, BAC-2, BAC-5). *AC - Anti-cell antibody patterns classified according to the International Consensus on ANA Patterns (ICAP)* [7]. *BAC - Brazilian anti-cell antibody patterns referring to transient code used for patterns not yet categorized by ICAP* [13]

**Fig. 4:**
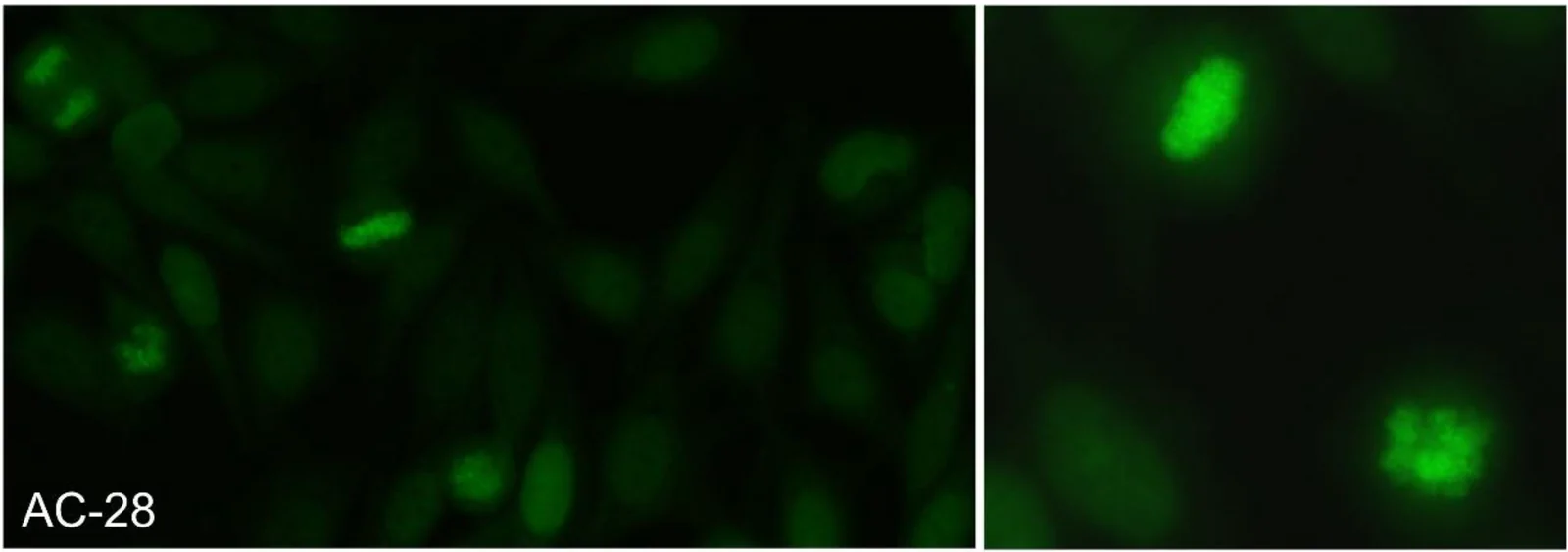
Morphological details of the Mitotic Chromosomal Pattern (AC-28). On the right side, a detailed view of the metaphase chromosomal plate and prophase is shown. Image credits from the International Consensus on ANA Patterns (Werner Klotz). (Microscope magnification: 400×)

The BCA maintained the difficulty level of the AC-2 pattern to the expert level, and added the AC-30 pattern to the same level, given their similar characteristics and the subtle nuances that must be recognized by the analyst to distinguish between them [22]. Consequently, at the competent level, the designation “nuclear speckled with positive chromosome metaphase plate” was introduced to encompass AC-2/AC-30 in cases where the analyst is unable to differentiate between the two patterns or the sample conditions do not allow for distinction. Figures 2 and 3 present the classification tree for users at the competent and expert levels, respectively, which can also be observed in Table 1.

## Inclusion of the nuclear myriad discrete speckled pattern (AC-31)

The BCA also incorporated the AC-31 pattern, designated “myriad discrete speckled,” into the classification tree. Previously, during the 6th ICAP Workshop in 2021, this pattern had been classified as AC-4a, a subgroup of the nuclear speckled with negative mitotic chromosome plate patterns group [23]. The AC-31 is strongly associated with anti-SS-A/Ro60 antibodies and is rarely observed in healthy individuals [24, 25]. Due to its association with anti-SS-A/Ro60 antibodies, representing an important marker for clinical investigation, ICAP assigned a specific code to this pattern, which is morphologically characterized by myriad discrete tiny nuclear dots and the absence of metaphase chromatin mass staining. As with any other pattern, the presumptive indication of anti-SS-A/Ro60 antibodies should always be confirmed using antigen-specific immunoassays [12, 24].

With respect to the clinical relevance, the AC-31 pattern contrasts with the AC-4 pattern in that AC-31 is strongly associated with anti-Ro60 antibodies, whereas the AC-4 pattern is frequently associated with no known autoantibodies and less frequently with SS-A/Ro60, SS-B/La, Mi-2, TIF1γ, TIF1β, and Ku. Accordingly, AC-4 is much more frequent than AC-31 in routine laboratory settings [12, 25].

The AC-31 pattern, like the AC-4 pattern, was considered an expert-level pattern in the classification tree due to the level of complexity required from the analyst in its identification [12]. Figure 1C and D show the morphological characteristics of the AC-31 and AC-4 patterns can be observed.

## Inclusion of the mitotic chromosomal pattern (AC-28)

The ICAP incorporated the AC-28 pattern into the classification tree during its first ICAP Workshop in 2014 [7]. This pattern is characterized by cells in prophase and metaphase showing chromosomal staining [7, 26, 27].

The AC-28 pattern has low positive predictive value for any specific disease [28] and is very rarely observed in a routine serology diagnostic setting [27]. The delay in the incorporation of the pattern by the BCA was due to its low frequency in laboratory routines and the limited information available regarding its antigenic identity. Reports indicate that the recognized antigens include DCA, MCA1, MCA4, and MCA5, with occurrences reported in diseases such as systemic lupus erythematosus, carcinoma, chronic lymphocytic leukemia, Sjögren’s disease, and polymyalgia rheumatica. However, specific immunoassays for these autoantibodies are not currently commercially available [26, 27, 29], and the true prevalence, clinical relevance, and autoantibody associations remain to be determined [7]. The AC-28 pattern was classified as competent-level and its morphological details are shown in Fig. 4.

## Inclusion of unclassified patterns (AC-XX)

The VII BCA incorporated into the classification tree the possibility of categorizing certain patterns as unclassified. According to Herold et al., unclassified patterns, coded as AC-XX, refer to transitional designation, meaning they have not yet been fully defined in terms of their morphological features and clinical relevance [30]. It is important to emphasize that these unusual or atypical patterns are not commonly observed in routine laboratory practice and should not be confused with complex patterns, which have already been previously detailed by the BCA [14]. The new ICAP classification tree presents a dedicated section for AC-XX unclassified patterns with a hyperlink that direct to images and information on several of these unusual patterns [31]. This should contribute to the training and recognition by analysts, as well as interpretation by attending physicians [12].

## Update on the classification tree with focus on the competent level patterns and guidance for laboratories on the classification of patterns in serum samples with low intensity reactivity

The VII BCA emphasized the importance of the competent-level classification by creating a competent-level tree exclusively with the mandatory patterns (Fig. 2). Considering the progressive improvement in the ability of Brazilian laboratories to recognize and classify patterns according to ICAP standards, the BCA recommended the inclusion of 22 mandatory patterns at competent level (Table 1). Within this competent-level tree, two distinct situations are identified. The first comprises a group of competent-level patterns that are readily identifiable due to their well-defined morphological features, allowing analyst to classify them primarily based on morphological criteria, for example, AC-3 (Centromere), AC-13 (PCNA-like), AC-22 (Polar speckled), among others. The second group consists of competent-level patterns that clustered two or more expert-level patterns according to their morphological similarities. This clustering reflects the challenges in the accurate distinction among these expert-level patterns in some situations, thereby allowing the analyst to elect a less assertive classification. These include, for instance, the nuclear speckled group with a negative metaphase chromosomal plate (AC-4/AC-5/AC-31), nuclear membrane patterns (AC-11/AC-12), cytoplasmic fibrillar patterns (AC-15/AC-16/AC-17), and cytoplasmic speckled patterns (AC-18/AC-19/AC-20). In such cases, analysts may lack sufficient experience to perform precise classification, or factors related to sample characteristics, such as low autoantibody concentration, may hinder proper pattern discrimination. For these scenarios, classification under the corresponding ‘umbrella’ category is recommended (Fig. 2, Table 1).

The competent-level pattern tree was revisited considering the limitations and challengeshistorically reported in the application of consensus recommendations to samples with low reactivity. A retrospective survey by Santos et al. (2022), found that more than half of the positive samples from a Brazilian laboratory had low HEp-2 IFA titers (1/80 or 1/160) [32]. Such a condition represents an additional challenge in characterizing the features of certain patterns due to the low fluorescence intensity, limiting the observation of essential morphological characteristics required for the classification of some patterns.

In these cases, the BCA recommends that the laboratory consider the classification at the competent level (Fig. 2) for such samples, also advising the inclusion of a note alerting about the limitations in defining patterns in low-reactivity samples. The VII consensus emphasizes that although some patterns demonstrate robustness in their morphological characteristics, as in the case of the AC-3 pattern, which remains characteristic even at low titers, several patterns may pose a significant challenge for the analyst under these conditions, suggesting they might be reported at the competent level. Figure 2 depicts the BCA classification tree with emphasis on the competent-level (mandatory) patterns with their respective encompassed expert-level patterns.

## Update of the classification tree at the expert level

The expert-level patterns were also revised with the to align with the recommendations of the VII ICAP [12]. In addition to the adjustments made to facilitate classification and interpretation at the national level, a review of the terminology in both English and Portuguese was conducted and made available on the official BCA website, www.HEp-2.com.br.

Regarding the nomenclature of composite patterns, which previously recommended a more detailed terminology [14] the VII Consensus adopted a more simplified approach, similar to that of ICAP, where the term ‘like’ was introduced for patterns named according to their associated autoantigens [12]. Table 1 lists the 27 patterns recognized at the expert level.

Modifications were also made to the layout of the classification tree to allow the grouping of patterns with similar morphological characteristics. For instance, the group of nuclear patterns with homogeneous or *quasi*-homogeneous fluorescence, along with positive chromosomal metaphase plate staining, was reorganized so that their boxes are placed in close proximity, reflecting their shared features. In this context, AC-1, BAC-3, and AC-29 were grouped together (Fig. 3). This arrangement is expected to facilitate pattern discrimination by analysts, especially when patterns exhibit overlapping morphological features that may lead to misinterpretation.

Patterns with similar morphological characteristics were grouped together, even if they belong to more than one classification group. This is the case for the composite pattern P-Ribosomal-like (BAC-5), which is classified as ‘Composite’ due to its reactivity against both the cytoplasm and nucleoli. However, because its cytoplasmic staining is highly prominent and resembles that of AC-19 pattern, the two were grouped together in the classification tree. This organization allows analyst to more easily compare and evaluate morphological similarities during interpretation. The 27 patterns at the expert level can be observed in the classification tree, each encompassed by a corresponding a competent-level pattern (Fig. 3).

**Table 1:** Pattern classification according to competence level, as recommended by the VII Brazilian consensus on autoantibodies

Competent-level patterns		Expert-level patterns	
Codes	Pattern designation	Codes	Pattern designation
AC-0	Negative		
AC-1/BAC-3/AC-29	Nuclear quasi-homogeneous to homogeneous	BAC-3	Nuclear *quasi*-homogeneous
		AC-29	Topo-I-like
AC-1	Nuclear homogeneous		
AC-2/AC-30	Nuclear speckled with positive chromosome metaphase plate	AC-2	Nuclear dense fine speckled
		AC-30	Nuclear fine speckled with mitotic plate
AC-3	Centromeric		
AC-4/AC-31/BAC-2/ AC-5/BAC-4	Nuclear speckled	AC-4	Nuclear fine speckled
		AC-31	Nuclear myriad discrete
		BAC-2	Ku-like
		AC-5	Nuclear coarse speckled
		BAC-4	Nuclear reticular coarse
AC6/AC-7	Nuclear discrete dots		
AC-6	Multiple nuclear dots		
AC-7	Few nuclear dots		
AC-8/AC-9/AC-10/BAC-1	Nucleolar	AC-8	Nucleolar homogeneous
		AC-9	Nucleolar clumpy
		AC-10	Nucleolar speckled
		BAC-1	RNA pol I/II-like
AC-11/AC-12	Nuclear membrane	AC-11	Nuclear membrane continuous
		AC-12	Nuclear membrane dotted
AC-13/AC-14 AC-13	Nuclear pleomorphic PCNA-like		
		AC-14	CENP-F-like
AC-15/AC-16/AC-17	Cytoplasmic fibrillar	AC-15	Cytoplasmic fibrillar linear
		AC-16	Cytoplasmic fibrillar filamentous
		AC-17	Cytoplasmic fibrillar segmental
AC-18/AC-19/BAC-5/AC-20	Cytoplasmic speckled	AC-18	Cytoplasmic speckled discrete dots
		AC-19	Cytoplasmic dense fine speckled
		BAC-5	P-Ribosomal-like
		AC-20	Cytoplasmic fine speckled
AC-21	Cytoplasmic speckled reticular		
AC-22	Cytoplasmic speckled polar		
AC-23	Cytoplasmic rods and rings		
AC-24	Mitotic centrosome		
AC-25/AC-26	Mitotic apparatus	AC-25	Mitotic fibers
		AC-26	NuMA-like
AC-27	Mitotic intercellular bridge		
AC-28	Mitotic chromosomal		
		Mixed / Multiple patterns	
		AC-XX (Unclassified)	

Legend: AC - Anti-cell antibody patterns classified according to the International Consensus on ANA Patterns (ICAP) [7]. BAC - Brazilian anti-cell antibody patterns referring to transient code used for patterns not yet categorized by ICAP [13]

## Final considerations

The VII Brazilian Consensus on Autoantibodies introduced important updates to the classification of immunofluorescence patterns in HEp-2 cells, with a particular emphasis on addressing the challenges posed by low-reactivity samples. The revision of the classification tree at both the competent and expert levels, in addition to incorporating the new patterns recently recognized by ICAP, aimed to ensure a more precise and standardized analysis and interpretation of the patterns based on their morphological characteristics. These updates also reflect the evolving technical capabilities of Brazilian laboratories in recognizing and classifying complex HEp-2 IFA patterns.

Additionally, with a focus on supporting less experienced users, the BCA included a simplified classification tree highlighting 22 patterns at the competent level, recommending its use in cases of serum samples with low fluorescence intensity or other circumstances that complicate expert-level classification. The terminology for composite patterns was also revised to enhance clarity and usability, while maintaining alignment with ICAP guidelines.

The implementation of these changes is intended to foster greater integration of Brazilian laboratories with international best practices, promoting higher uniformity, reproducibility, and clarity in autoantibody reporting. These improvements are expected to significantly enhance the quality of laboratory analysis and reflect the growing proficiency of professionals in applying the criteria required for accurate pattern characterization. Importantly, while aligned with ICAP, the VII BCA supports the implementation and training of Brazilian laboratories, taking into account the country’s specific characteristics.

## Electronic supplementary material

Below is the link to the electronic supplementary material.

Supplementary Material 1

## Data Availability

No datasets were generated or analysed during the current study.

## Abbreviations

ANA: Antinuclear Antibody
BAC: Brazilian anti-cell autoantibodies
BCA: Brazilian Consensus on ANA
FS/non-DFS: Fine speckled/non-Dense Fine Speckled
HEp-2: Human epithelial type 2
ICAP: International Consensus on ANA Patterns
IFA: Indirect Immunofluorescent Assay
MCAs: Mitotic Chromosomal Autoantigens
NuMA: Nuclear Mitotic Apparatus
PCNA: Proliferating Cell Nuclear Antigen
CENP-F: Centromere-Associated Protein F
