# Supplementary material for: VII Brazilian Consensus guidelines for the detection and interpretation of anti-cell autoantibodies on HEp-2 cells
Source: Adv Rheumatol. 2025 Nov 25;65:61. doi: 10.1186/s42358-025-00493-0 (PMC13488776; doi:10.1186/s42358-025-00493-0)
Supplement: Supplementary file 1 [file 42358_2025_493_MOESM1_ESM.docx]

**Table 1**
Pattern classification by competence level, as recommended by the VII Brazilian Consensus on Autoantibodies (BCA), with nomenclature in Portuguese.

| **Padrões do nível competente** | | **Padrões do nível especialista** | |
| --- | --- | --- | --- |
| **Código** | **Designação do padrão** | **Código** | **Designação do padrão** |
| **AC-0** | Negativo |  |  |
| **AC-1/BAC-3/AC-29** | *Quasi*-homogêneo a homogêneo | **BAC-3** | Nuclear *quasi*-homogêneo |
| **AC-1** | Nuclear homogêneo | **AC-29** | Topo-I-like |
| **AC-2/AC-30** | Nuclear pontilhado com PMC corada | **AC-2** | Nuclear pontilhado fino denso |
|  |  | **AC-30** | Nuclear pontilhado fino com placa |
| **AC-3** | Centromérico |  |  |
| **AC-4/AC-31/BAC-2/AC-5/BAC-4** | Nuclear pontilhado | **AC-4** | Nuclear pontilhado fino |
|  |  | **AC-31** | Nuclear pontilhado fino de pontos distintos |
|  |  | **BAC-2** | Ku-like |
|  |  | **AC-5** | Nuclear pontilhado grosso |
|  |  | **BAC-4** | Nuclear pontilhado grosso reticulado |
| **AC6/AC-7** | Pontos nucleares isolados |  |  |
| **AC-6** | Múltiplos pontos isolados |  |  |
| **AC-7** | Raros pontos isolados |  |  |
| **AC-8/AC-9/AC-10/BAC-1** | Nucleolar | **AC-8** | Nucleolar homogêneo |
|  |  | **AC-9** | Nucleolar aglomerado |
|  |  | **AC-10** | Nucleolar pontilhado |
|  |  | **BAC-1** | RNA polimerase I/II-like |
| **AC-11/AC-12** | Membrana nuclear | **AC-11** | Membrana nuclear contínua |
|  |  | **AC-12** | Membrana nuclear pontilhada |
| **AC-13/AC-14** | Nuclear pleomórfico | **AC-14** | CENP-F-like |
| **AC-13** | PCNA-like |  |  |
| **AC-15/AC-16/AC-17** | Citoplasmático fibrilar | **AC-15** | Citoplasmático fibrilar linear |
|  |  | **AC-16** | Citoplasmático fibrilar filamentoso |
|  |  | **AC-17** | Citoplasmático fibrilar segmentar |
| **AC-18/AC-19/BAC-5/AC-20** | Citoplasmático pontilhado | **AC-18** | Citoplasmático pontilhado de pontos isolados |
|  |  | **AC-19** | Citoplasmático pontilhado fino denso |
|  |  | **BAC-5** | P-Ribossomal-like |
|  |  | **AC-20** | Citoplasmático pontilhado fino |
| **AC-21** | Citoplasmático pontilhado reticular |  |  |
| **AC-22** | Citoplasmático pontilhado polar |  |  |
| **AC-23** | Citoplasmático em anéis e bastões |  |  |
| **AC-24** | Centrosomo |  |  |
| **AC-25/AC-26** | Aparelho mitótico | **AC-25** | Fuso mitótico |
|  |  | **AC-26** | NuMA-like |
| **AC-27** | Ponte intercelular |  |  |
| **AC-28** | Cromossomos mitóticos |  |  |

**Fig. 1**
BCA classification tree in Portuguese, highlighting the competent-level patterns. AC - Anti-cell antibody patterns classified according to the International Consensus on ANA Patterns (ICAP) [7]. BAC - Brazilian anti-cell antibody patterns referring to transient code used for patterns not yet categorized by ICAP [13]

**Fig. 2**

BCA classification tree in Portuguese, highlighting the expert-level patterns (optional report). Each expert-level pattern is nested within a corresponding competent-level pattern, allowing the analyst to choose between the expert- and competent-level designations based on the characteristics observed in each sample image. An asterisk indicates that the pattern belongs to the composite group and is therefore not linked to a single main group (AC-14, AC-26, AC-29, BAC-1, BAC-2, BAC-5). *AC - Anti-cell antibody patterns classified according to the International Consensus on ANA Patterns (ICAP)* [7]. *BAC - Brazilian anti-cell antibody patterns referring to transient code used for patterns not yet categorized by ICAP* [13].
